# Unsupervised Machine-Learning-Based Endotype Discovery Using Iterative Resampling in Dupilumab-Treated Patients

**DOI:** 10.3390/ijms27125266

**Published:** 2026-06-10

**Authors:** Emma Moreno-Jiménez, Natalia Morgado, Asunción García-Sánchez, Juan Carlos Triviño, Miguel Estravís, Manuel Gómez-García, María Gil-Melcón, Milagros Lázaro-Sastre, Catalina Sanz, María Isidoro-García, Ignacio Dávila

**Affiliations:** 1 Departamento de Microbiología y Genetica, Universidad de Salamanca, 37007 Salamanca, Spain; emmamoreno@usal.es (E.M.-J.); nmorgado@usal.es (N.M.); estravis@usal.es (M.E.); 2 Instituto de Investigación Biomédica de Salamanca, 37007 Salamanca, Spain; chonela@usal.es (A.G.-S.); mgomezgarcia4.ibsal@saludcastillayleon.es (M.G.-G.); milagroslazaro@saludcastillayleon.es (M.L.-S.); misidoro@saludcastillayleon.es (M.I.-G.); idg@usal.es (I.D.); 3 Instituto de Salud Carlos III, Red de Enfermedades Inflamatorias—RICORS, 28029 Madrid, Spain; 4 Departamento de Biomedicina y Ciencias del Diagnostico, Universidad de Salamanca, 37007 Salamanca, Spain; 5 Synlab Group, Sistemas Genómicos SL, 46980 Paterna, Spain; jc.trivino@sistemasgenomicos.com; 6 Servicio de Bioquímica Clínica, Hospital Universitario de Salamanca, 37007 Salamanca, Spain; 7 Servicio de Otorhinolaringología y Cirugía de cabeza y cuello, Hospital Universitario de Salamanca, 37007 Salamanca, Spain; mgilmel@saludcastillayleon.es; 8 Servicio de Aergología Department, Hospital Universitario de Salamanca, 37007 Salamanca, Spain; 9 Departamento de Medicina, Universidad de Salamanca, 37007 Salamanca, Spain

**Keywords:** severe asthma, CRSwNP, dupilumab, transcriptomics, endotypes, super-response, precision medicine

## Abstract

Asthma is a heterogeneous inflammatory disorder involving multiple immune pathways, frequently presenting alongside comorbidities such as chronic rhinosinusitis with nasal polyps (CRSwNP). Although biologic therapies such as dupilumab have shown clinical efficacy, the molecular mechanisms underlying variable treatment responses remain poorly understood. This study aimed to characterize transcriptomic patterns that distinguish asthmatic patients from healthy controls and to evaluate transcriptomic changes induced by dupilumab. Whole-blood RNA-seq was performed in 66 samples, 18 patients (G0) with severe asthma before and after 6 months of dupilumab treatment compared with 30 non-asthmatic controls. Differentially expressed genes (DEGs) were identified and validated by quantitative PCR (qPCR). Clinical responses were assessed using the FEV1, Exacerbations, Oral corticosteroids, Symptoms (FEOS) score and the Sino-Nasal Outcome Test-22 (SNOT-22). A total of 1124 DEGs were identified, distinguishing asthmatic patients from controls. Notably, *ABCC1*, *CYP4F12*, *FBN1*, *IKZF2*, and *RAB44* were differentially expressed across all patients’ subgroups and are proposed as putative general disease biomarkers. Unsupervised machine learning analysis of pre- vs. post-dupilumab transcriptomic profiles identified two distinct patient subgroups within G0, here termed G1 and G2. When comparing baseline vs. post-treatment samples in the overall cohort (G0), only 12 DEGs were identified. In contrast, stratified analysis revealed 1288 DEGs in G1 and 354 DEGs in G2, suggesting divergent molecular response to treatment. Additionally, baseline expression of *DIXDC1* was identified as a predictor of CRSwNP non-super-responders. By applying unsupervised machine learning to transcriptomic profiles, this exploratory study identifies two distinct endotypes with divergent molecular mechanisms of response to dupilumab, supporting a precision medicine approach to biologic therapy in severe asthma.

## 1. Introduction

The upper and lower airways share pathophysiological mechanisms with implications for the management of respiratory comorbidities. Thus, chronic rhinosinusitis with nasal polyps (CRSwNP) and asthma are closely associated and frequently coexist [1]. Additionally, non-steroidal anti-inflammatory drug (NSAID)-exacerbated respiratory disease (N-ERD) is a chronic eosinophilic inflammatory disorder that occurs in patients with asthma and CRSwNP and is frequently associated with a worse evolution [2].

The recognition of distinct asthma endotypes has improved the understanding of disease heterogeneity and therapeutic responses [3]. Three main inflammatory patterns have been identified [4,5,6,7]: type 2-high, type 2-low, and mixed endotypes, which may share overlapping genetic, epigenetic, metabolic, neurogenic, and remodeling pathways [3,8,9,10]. Biomarkers, including blood or sputum eosinophils, FeNO, serum periostin, total and specific IgE, epithelial or sputum cell gene expression profiles, and saliva inflammatory signatures, are currently used to phenotype and endotype asthma and CRSwNP [11,12]. However, none fully meets the criteria for an ideal biomarker—namely, pathway-specific, reproducible, and cost-effective—highlighting the need for improved patient characterization to optimize personalized treatment [12,13,14,15].

Dupilumab is a fully human monoclonal antibody that targets the IL-4Rα subunit, inhibiting signaling of both IL-4 and IL-13, two central cytokines in type 2 inflammation. In 2018, the FDA approved dupilumab for the treatment of moderate-to-severe asthma in patients with blood eosinophils greater than 150 cells/μL or FeNO greater than 25 ppb [16]. It is also indicated for the treatment of atopic dermatitis (AD), chronic spontaneous urticaria, eosinophilic esophagitis (EoE), chronic obstructive pulmonary disease (COPD), CRSwNP, bullous pemphigoid, and prurigo nodularis in adults [17,18,19,20]. Its dual efficacy in both upper and lower airway disease highlights its role as a targeted therapy for patients with severe type-2-driven respiratory disorders [21,22,23,24]. The cost of dupilumab, together with the lack of response in roughly a quarter of patients [25], underscores the importance of identifying predictive biomarkers of treatment response.

This prospective study sought to delineate gene expression signatures associated with asthma and type 2 comorbidities and to characterize clinical responses to dupilumab by employing machine learning approaches. Such insights enable precise stratification of patients who are most likely to benefit from this therapy, thereby advancing personalized asthma management.

## 2. Results

### 2.1. Study Population and Response to Treatment

Demographic and clinical characteristics of all participants are summarized in Table 1. Our cohort showed differences in IgE levels and eosinophil counts between controls and patients, but not in sex or age. At T0, patients in different clinical subgroups exhibited different levels of FEV_1_, FeNO, and SNOT-22; those with N-ERD had higher FEV_1_ and SNOT-22 values than asthmatic patients with CRSwNP (Supplementary Table S1).

After treatment with dupilumab, patients experienced statistically significant improvements in symptoms and quality of life (QoL) and decreases in OCS use, annualized rate of severe exacerbations, SNOT-22 score, FeNO, and serum IgE levels. Moreover, there was a significant increase in ACT scores and lung function (FEV_1_), as shown in Table 1. When applying the criteria for super-response (SR), 13/18 patients showed asthma SR by FEOS index and 4/16 showed CRSwNP SR by SNOT-22 criteria (due to missing data in two patients) (Table 2).

### 2.2. Transcriptomic Profile in Asthmatics and Controls

**Figure 1:**
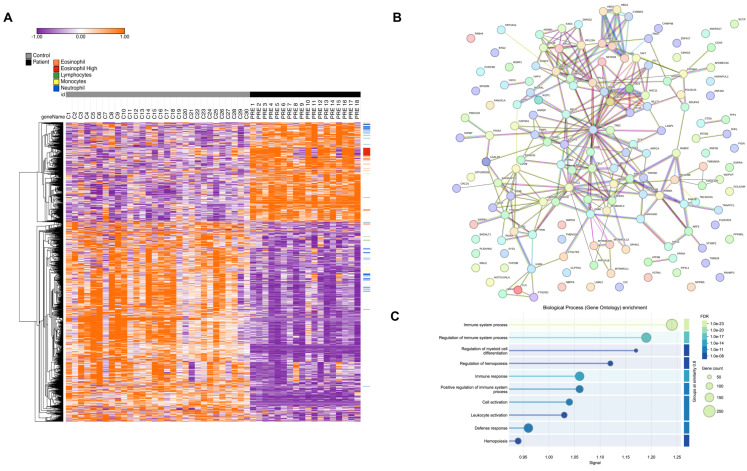
Transcriptomic variations observed between non-asthmatic controls and asthmatic patients. (**A**) Heatmap depicting the expression levels of differentially expressed genes (DEGs) between 30 controls and 18 patients. The upper grey bar represents the gene expression levels of healthy controls, while the upper black bar represents those of patients. The right-side bar highlights Pearson correlations between gene expression and peripheral blood cell counts obtained from complete blood count (CBC) with differential: red indicates genes highly correlated with peripheral blood eosinophil counts (R > 0.7), orange for moderate correlation with peripheral blood eosinophil counts (0.5 < R < 0.7), green for correlation with total peripheral blood lymphocyte counts (0.5 < R < 0.7), blue for correlation with peripheral blood neutrophil counts (0.5 < R < 0.7), and yellow for correlation with peripheral blood monocyte counts (0.5 < R < 0.7). (**B**) The protein–protein interaction network of the study’s top 200 DEGs, sorted by padj, was generated using the STRING protein interaction database. (**C**) STRING functional enrichment analysis of Biological Processes.

Comparing basal blood RNA-seq profiles from asthmatic patients with controls, we identified 1124 differentially expressed genes. Of these, 380 showed increased expression, while 744 showed decreased expression in patients. Among the identified genes, 958 were protein-coding, three corresponded to immunoglobulin constant region genes, and 163 were unannotated (Figure 1A, Supplementary Table S2).

A correlation analysis was performed with transcriptomic data and CBD/differential count of the peripheral blood, finding 34 DEGs highly correlated with peripheral blood eosinophil counts (R > 0.7), 18 with a moderate correlation (0.7 ≤ R > 0.5) with eosinophil counts, 51 correlated with peripheral blood neutrophil counts (0.7 ≤ R > 0.5), seven correlated with peripheral blood monocyte counts (0.7 ≤ R > 0.5) and three correlated with total peripheral blood lymphocyte counts (0.7 ≤ R > 0.5) (Figure 1A, Supplementary Table S2).

STRING analysis (v12) of protein–protein interactions revealed strong connectivity among these DEGs (*p*-value < 1 × 10^−16^) (Figure 1B). Gene Ontology (GO) enrichment for biological processes showed that DEGs were mainly associated with regulation of immune system processes and immune and defence responses (Figure 1C, Supplementary Table S3).

Over-representation analysis GO slim terms (Supplementary Table S4) and GSEA for GO terms (Supplementary Table S5) highlighted processes related to cytokine-mediated signaling and immune-response-regulating signaling pathways.

### 2.3. Transcriptomic Profile and Analysis of Putative Biomarkers of Response to Dupilumab

**Figure 2:**
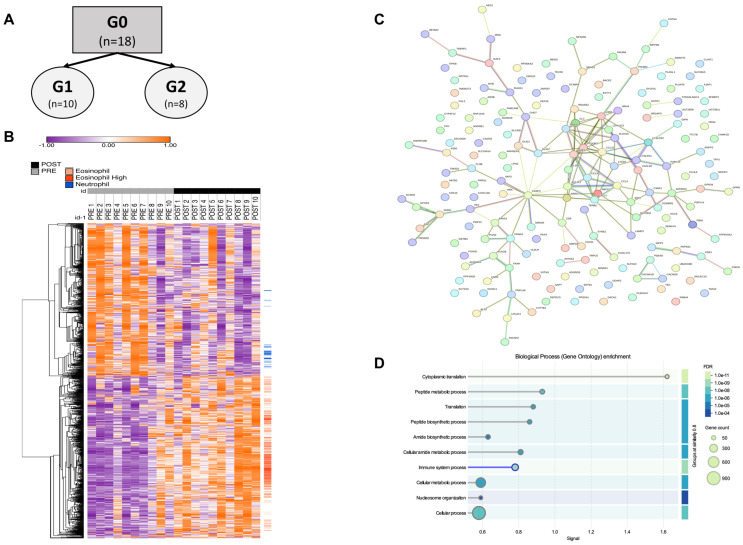
Transcriptomic variations observed following treatment with dupilumab. (**A**) Diagram of subgroups’ transcriptomic distribution illustrating the explicit subdivision of the overall patient cohort (G0, *n* = 18) into two distinct transcriptomic subgroups, G1 (*n* = 10) and G2 (*n* = 8), following dupilumab treatment; *n*, number of samples. (**B**) Heatmap depicting the expression levels of differentially expressed genes (DEGs) before and after treatment in 10 patients (G1). The upper grey bar represents gene expression levels pre-treatment, while the upper black bar represents post-treatment expression levels. The right-side bar highlights Pearson correlations between gene expression and peripheral blood cell counts obtained from complete blood count (CBC) with differential: red indicates genes highly correlated with peripheral blood eosinophil counts (R > 0.7), orange for moderate correlation with peripheral blood eosinophil counts (0.5 < R < 0.7), and blue for correlation with peripheral blood neutrophil counts (0.5 < R < 0.7). (**C**) The protein–protein interaction network of the study’s top 200 DEGs, sorted by padj, was generated using the STRING protein interaction database. (**D**) STRING functional enrichment analysis of Biological Processes.

Initially, RNA-seq analysis of the whole cohort (G0) revealed only 12 genes with significant differential expression. This limited yield is consistent with the well-documented signal dilution effect that occurs when heterogeneous patient populations are analyzed together: patients with divergent transcriptional profiles are pooled, reducing statistical power and masking subgroup-specific signals. Consequently, genes that are strongly differentially expressed in a subset of patients may not reach significance when the full cohort is tested, while the same genes can emerge robustly when the analysis is restricted to a more transcriptionally homogeneous subgroup (Supplementary Table S6). The unsupervised machine learning approach allows addressing this heterogeneity, identifying potential patient subpopulations with distinctive transcriptional profiles. Two combinatorial subgrouping strategies were directly applied to the expression data, evaluating all possible patient partitions across multiple group sizes without using any clinical variables [27,28]. The analysis revealed two statistically significant subgroups, designated G1 and G2, in which G1 exhibited a robust differential expression signature (Figure 2A). Remarkably, these subgroups were eventually characterized by distinct eosinophil, basophil, and lymphocyte patterns (Table 3).

Interestingly, in the G1 subgroup, the comparison of pre-treatment (T0) and post-treatment (T6) revealed significant differences in the expression of 1288 genes (Figure 2B, Supplementary Table S7). Of these, 662 exhibited increased expression levels, while 626 showed decreased expression after treatment.

The correlation analysis between gene expression and CBD/differential count of the peripheral blood revealed that 117 DEGs highly correlated with peripheral blood eosinophil counts (R > 0.7), 254 had a moderate correlation (0.7 ≤ R > 0.5) with peripheral blood eosinophil counts, and 43 were correlated with peripheral blood neutrophil counts (0.7 ≤ R > 0.5) (Figure 2B, Supplementary Table S7).

STRING analysis (v12) of protein–protein interactions revealed strong connectivity among these DEGs (*p*-value = 1.55 × 10^−15^) (Figure 2C). GO enrichment for biological processes showed that DEGs were particularly associated with cytoplasmic translation and cellular, metabolic, and immune processes (Figure 2D, Supplementary Table S8).

Over-representation analysis GO slim terms (Supplementary Table S9) and GSEA for GO terms (Supplementary Table S10) showed processes related to cytokine signaling and transcriptomic regulation.

Regarding the G2 subgroup, we found 358 DEGs after treatment: 215 exhibited increased expression, while 143 showed decreased expression (Supplementary Table S11).

**Figure 3:**
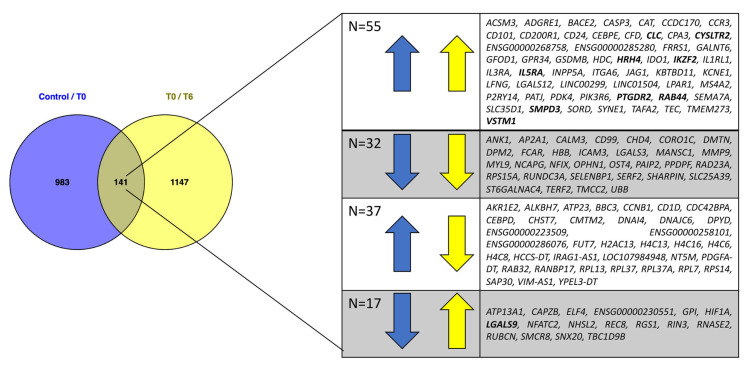
Common DEGs in both transcriptomic studies. Venn diagram illustrating common DEGs identified in transcriptomic comparisons (Healthy Controls (*n* = 30) vs. Asthmatic Patients (G0, *n* = 18)); Patients before dupilumab treatment vs. Patients six months after treatment (G1, *n* = 10). Upward arrows (↑) indicate genes upregulated in asthmatic patients or after six months of dupilumab treatment (T6), whereas downward arrows (↓) indicate downregulated genes in the same comparisons; *n*, number of samples.

Otherwise, when comparing controls vs. patients and pre- vs. post-treatment, we identified 141 common genes. Fifty-five genes were overexpressed, 32 were underexpressed, and 54 showed opposite regulation between the two comparisons. Among the 20 genes with the highest padj values, in the HCs vs. patient comparison, *CLC* was upregulated in patients, whereas in the pre- vs. post-treatment analysis, *IKZF2*, *CYSLTR2*, and *PTGDR2* were upregulated after treatment (Figure 3).

### 2.4. Validation Analysis of Disease and Response to Dupilumab Treatment Biomarkers

After transcriptomic analysis, we evaluated changes in the relative expression levels of 23 genes selected for their relevance to type 2 inflammatory processes and to changes observed in our RNA-seq dataset, using quantitative PCR (qPCR) (Table 4).

When comparing the relative expression of these genes between controls and patients, significant differences were observed for *ABCC1*, *CYP4F12*, *FBN1*, *IKZF2*, and *RAB44* in the general population (G0) and in the G1 and G2 patient subgroups (Table 4, Supplementary Figure S1A). There were also other differentially expressed genes in G1 and G2 subgroups (Table 4, Supplementary Figure S2).

Regarding comorbidities, there was differential expression of *LGALS9* in asthma with CRSwNP and N-ERD (AUC: 0.833, IC 95% = 0.639–1.000, *p*-value = 0.001), with higher expression in N-ERD patients, allowing differentiation between the two patients’ groups (Supplementary Figure S1B).

In the pre- vs. post-treatment validation study in patients treated with dupilumab, results considering all patients together (G0) are shown in Table 4, Supplementary Figure S3. Additionally, in the G1 subgroup, we observed statistically significant differences for several genes, whereas in the G2 subgroup, significance was detected only for *SETD1B* (Table 4, Supplementary Figure S4).

### 2.5. Analysis of the Response to Dupilumab Treatment

Patient outcomes were evaluated using the FEOS index [26] (Supplementary Table S12) and SNOT-22 score [29] and classified as SRs or non-SRs (Table 2).

**Figure 4:**
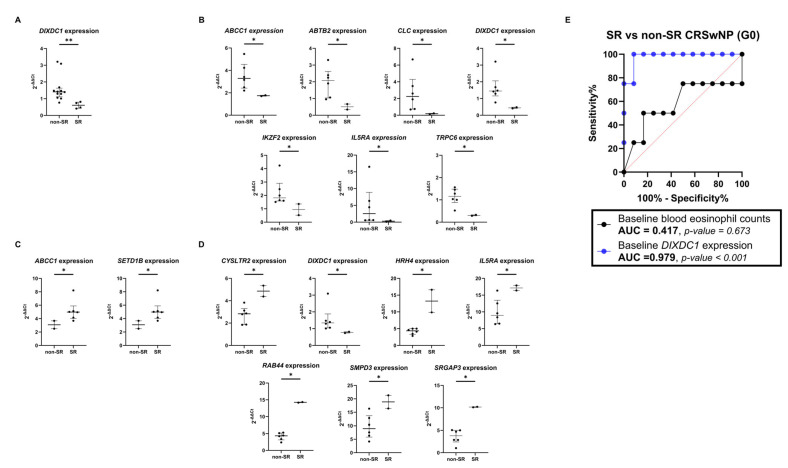
Baseline differential gene expression by response to asthma and CRSwNP symptoms or dual super-response after dupilumab treatment, and ROC curve for CRSwNP SR or non-SR predictive value. (**A**) Baseline gene expression levels were measured by qPCR comparing CRSwNP non-SR or SR in the total cohort of patients (*n* = 18). (**B**) Baseline gene expression levels were measured by qPCR comparing CRSwNP non-SR or SR in the G1 subgroup of patients (*n* = 10). (**C**) Baseline gene expression levels were measured by qPCR comparing asthma non-SR or SR in the G2 subgroup (*n* = 8). (**D**) Baseline gene expression levels were measured by qPCR comparing CRSwNP non-SR or SR in the G2 subgroup of patients (*n* = 8). (**E**) ROC curve illustrating the predictive value of baseline eosinophil counts and *DIXDC1* for classifying patients as CRSwNP SR or non-SR; Area under the curve (AUC) values and *p*-value are indicated (*, *p* < 0.05; **, *p* < 0.01, Wilcoxon test or Mann–Whitney test). The red dotted line represents the reference line corresponding to random classification (AUC = 0.5).

When analyzing putative response genetic biomarkers for CRSwNP, the G0 group showed a statistically significant difference in *DIXDC1* baseline expression (*p*-value = 0.005), with higher levels in non-SR (1.408 (1.115, 1.627)) vs. SR (0.609 (0.425, 0.796)) (Figure 4A).

Considering patients’ subgroups, we found that, in the G1 subgroup, basal expression of *ABCC1*, *ABTB2*, *CLC*, *DIXDC1*, *IKZF2*, *IL5RA*, and *TRPC6* could also differentiate between SR and non-SR in CRSwNP patients (*p*-value = 0.046 for all), with higher expression in non-SR patients (Figure 4B).

In the G2 subgroup, we detected significantly higher basal expression of *ABCC1* and *SETD1B* in asthma SRs compared with non-SRs (*p*-value = 0.046 for both genes) (Figure 4C). Regarding CRSwNP SR, we observed higher *CYSLTR2*, *HRH4*, *IL5RA*, *RAB44*, *SMPD3* and *SRGAP3* expression in SRs (*p*-value = 0.046 in all cases) and higher *DIXDC1* expression in non-SRs (*p*-value = 0.046) (Figure 4D). All patients who required a change in treatment or experienced adverse events exhibited basal elevated expression levels.

The area under the curve (AUC) indicated that basal eosinophil counts were an unreliable predictor of CRSwNP super-response (AUC: 0.417, IC 95% = 0.029–0.804, *p*-value = 0.673). However, *DIXDC1* basal expression was a good predictor of CRSwNP non-SR in G0 (AUC: 0.979, IC 95% = 0.917–1.000, *p*-value < 0.001) (Figure 4E). Furthermore, in both subgroups (G1 and G2), the ROC curve showed that each gene individually reached an AUC of 1.0 (*p*-value < 0.001), and the combined gene set also showed an AUC of 1.0, reflecting complete separation within the available sample.

## 3. Discussion

Our study confirms that dupilumab significantly improved clinical outcomes in patients with severe asthma, as evidenced by reductions in oral corticosteroid use and annual exacerbations, improvements in lung function (FEV_1_) and asthma control, and lower SNOT-22 scores. These findings align with clinical trials and real-world evidence in asthma [11,30,31,32] and CRSwNP [33,34]. Its dual efficacy in upper and lower airway disease highlights its role as a targeted therapy for patients with severe type-2-driven respiratory disorders [21,22,23,24]. Notwithstanding, the cost of dupilumab, together with the lack of response in some patients, underscores the importance of identifying predictive biomarkers of treatment response.

RNA-seq analysis of controls and asthmatic patients identified 1124 DEGs associated with diverse immune cell types, reflecting a coordinated activation of both innate and adaptive immune pathways. Furthermore, the strong connectivity observed among these DEGs suggests a tightly coordinated transcriptional network linked to immune regulation, inflammatory processes, and defense responses [35,36], consistent with findings in the U-BIOPRED Cohort [37]. These results indicate that healthy individuals maintain a balanced, tightly regulated immune network, whereas patients exhibit signs of dysregulated or suppressed immune activity.

Although *LGALS9* (galectin-9) promotes Foxp3^+^ Treg differentiation and stability, enhancing suppressive function [38], its increased expression in N-ERD could reflect a compensatory response to the more pronounced type 2 inflammatory environment.

A central finding of this study was the identification of two distinct molecular trajectories in response to dupilumab (G1 and G2). When analyzing the whole cohort, molecular differences were masked (yielding only 12 DEGs) as observed in other studies [39,40]. It is noteworthy that unsupervised clustering effectively unveiled this underlying heterogeneity, suggesting that these subgroups represent distinct biological endotypes [34,41,42]. Furthermore, the G1 and G2 groups showed differences in peripheral blood populations between baseline and 6 months post-treatment. This difference in cell populations may result from blockade of the IL-4/IL-13 pathway, which may prevent the selective recruitment of inflammatory cells to the tissue [43].

It seems that there are at least two modes of response to IL-4/IL-13 blockade. These differences were reinforced by subgroup-specific transcriptomic responses. The G1 subgroup showed a robust genomic response to dupilumab, with 1288 DEGs, whereas G2 showed a more limited signature (358 DEGs, none of which reached padj < 0.05). This suggests that G1 patients experienced a broader shift in gene expression upon inhibition of the IL-4/IL-13. In G1, DEGs were mainly associated with eosinophil and neutrophil activity and were enriched for metabolic and immune processes, consistent with dupilumab’s immunomodulatory effects on type 2 inflammation [31]. These findings are biologically plausible, as IL-4/IL-13 blockade specifically suppresses type 2 inflammation but, in these patients, may not be the principal modification and could enhance alternative inflammatory pathways. Moreover, changes in immune cell composition induced by dupilumab and early transitional responses during immune rebalancing may contribute to this transcriptional increase [44]. However, in G2, we could not observe significant transcriptional changes, reinforcing the idea of two distinct endotypes.

To translate these findings into practical biomarkers, we validated 23 genes by qPCR in non-asthmatic controls and patients, chosen for their biological relevance and notable changes in our RNA-seq data. The comparison of asthmatic patients and healthy controls in peripheral blood has been extensively reported in previous transcriptomic studies. In agreement with prior work, we observed differential expression of genes previously associated with asthma-related immune dysregulation [41,42,45,46,47]. Notably, several of the identified genes overlap with established asthma blood transcriptomic signatures including genes related to eosinophilic activation, type 2 immune signaling, and airway remodeling previously described in those studies, supporting the validity and robustness of our dataset. Our findings reveal several genes previously characterized in diverse biological contexts, such as *ABCC1*, in airway smooth-muscle relaxation through cAMP transport [48]; *CYP4F12*, in lipid metabolism and eosinophil function [49]; *FBN1*, in RNA-splicing defects [50]; *IKZF2*, a key transcription factor for regulatory T-cell stability [51]; and Rab family members, including *RAB44* in vesicular trafficking and immune regulation [52] that were significantly dysregulated in our patient cohort compared with controls, suggesting previously unreported involvement in asthma pathophysiology. By integrating these established functional roles with our differential expression data, the present study provides new evidence that these pathways may be actively dysregulated in disease, thereby highlighting their potential mechanistic roles.

Importantly, the novelty of our study does not reside in the case–control comparison per se but in the integration of this baseline transcriptomic information with post-treatment data and the correlation with clinical data, all of this together allowing the identification of distinct patient subgroups (G1 and G2) through unsupervised machine learning. This stratification revealed heterogeneity in gene expression patterns that is not captured in traditional case–control comparisons and may help explain differential molecular responses to treatment. Regarding changes in relative gene expression in patients after dupilumab treatment, we identified that the histone methyltransferase *SETD1B* was the only downregulated gene across all subgroups, suggesting a shared epigenetic mechanism of action. Notwithstanding, G1 patients exhibited broad transcriptomic changes involving key type 2 mediators (e.g., *IL5RA*, *CLC*) and metabolic mediators. Still, we did not observe these transcriptomic changes in G2, validating distinct endotypes with divergent transcriptional plasticity under IL-4Rα blockade. Dupilumab alters the IL-4/IL-13 signaling environment, modifying immune cell activation states and distribution. Increased eosinophil-related transcripts may reflect circulating eosinophils [53] rather than tissue infiltration [54], while matrix- and metabolism-associated genes likely correspond to epithelial restoration [55]. Induction of chromatin-remodeling and methylation genes may suggest epigenetic reprogramming that stabilizes inflammation resolution [56]. Collectively, the observed transcriptional shifts might support the notion that dupilumab fosters immune recalibration rather than suppression, promoting a controlled reparative environment associated with clinical improvement. Stratified analysis revealed residual Th2-related activity in G1, possibly reflecting eosinophil redistribution or compensatory IL-5/prostaglandin signaling [57,58], and a different expression pattern in G2. Despite these differences, both groups achieved comparable clinical benefit, supporting the concept that dupilumab efficacy reflects immune recalibration rather than eosinophil depletion [59].

Regarding the response to dupilumab treatment, *DIXDC1* basal expression appeared to be a potential predictor of limited response. It is a positive regulator of Wnt/β-catenin signaling and a modulator of cytoskeletal dynamics and was consistently identified in CRSwNP non-SR. Its persistent expression in partial responders suggests ongoing epithelial–mesenchymal-transition-like programs, structural remodeling, or aberrant repair mechanisms that are not directly targeted by IL-4Rα blockade. In addition, Wnt/β-catenin signaling has been implicated in barrier dysfunction, fibroblast activation, and extracellular matrix deposition in chronic airway disease [60,61], supporting the notion that dupilumab-refractory disease may be driven by remodeling pathways that operate independently of type 2 inflammation. Interestingly, all patients who either required a change in treatment or experienced adverse events exhibited elevated expression levels in the pre-treatment condition. Therefore, *DIXDC1* may serve as a predictive biomarker of CRSwNP non-SR and point to alternative pathogenic mechanisms that warrant adjunctive therapeutic strategies. The near-perfect AUC observed for *DIXDC1* should be interpreted with caution, as it is likely influenced by the small sample size and the high dimensionality of the data, which increase the risk of overfitting. Therefore, this result should be considered exploratory rather than definitive. Independent validation in larger cohorts will be necessary to assess the true predictive performance of *DIXDC1*.

Study limitations include a relatively small sample size and the single-center design, which may limit generalizability. In particular, subgroup analyses increase the risk of false-positive findings and limit statistical robustness. Nevertheless, the samples included in this study were carefully selected according to stringent quality and clinical criteria in order to minimize potential sources of variability and ensure the reliability of the differential expression analysis. In addition, the use of clinically matched paired samples provides a more robust and biologically reliable comparison than conventional analyses based on unrelated or randomly selected samples, as it reduces inter-individual variability and better captures disease-associated molecular differences. Moreover, the six-month follow-up period may not fully capture long-term transcriptional adaptations to dupilumab, and the use of peripheral blood as the sole biological matrix may not fully reflect local mucosal and epithelial transcriptomic changes in the airways. Given the exploratory nature of these analyses and the limited sample size in certain groups, these results should be confirmed in larger populations. However, despite these constraints, the study provides exploratory insights into systemic immune modulation associated with dupilumab treatment.

## 4. Materials and Methods

### 4.1. Study Population

This study involved 66 samples, 36 corresponding to 18 asthmatic patients pre- and post-treatment with dupilumab (6 with asthma and CRSwNP, and 12 with N-ERD) and 30 corresponding to healthy controls (HCs) from the Multidisciplinary Asthma Unit of the Allergy, Respiratory, and Otorhinolaryngology Departments of the University Hospital of Salamanca who met the following inclusion criteria. Controls fulfilled the following criteria: (i) no symptoms or history of asthma or other pulmonary diseases, (ii) no symptoms or history of rhinitis, (iii) no symptoms or history of allergic diseases, (iv) negative skin prick tests to a battery of common aeroallergens, (v) absence of a family history of asthma, rhinitis, or atopy, and (vi) age > 16 years. Age was significantly higher in controls than in patients in order to permit a more extended period for asthma to have appeared. In addition, patients meeting the following criteria were recruited: (i) physician diagnosis of respiratory allergy (asthma, rhinitis, nasal polyposis or aspirin-exacerbated respiratory disease (N-ERD)), (ii) age > 16 years. Asthma was diagnosed according to GINA guidelines [62], and its phenotype classification was determined using the Spanish Guide for the Management of Asthma (GEMA 5.5) [63]. Severe uncontrolled asthma was diagnosed using the American Thoracic Society (ATS) and European Respiratory Society (ERS) criteria [64,65]. CRSwNP and N-ERD were diagnosed according to the European Position Paper on Rhinosinusitis and Nasal Polyps (EPOS) clinical diagnostic criteria [66]. Skin prick tests were also conducted as previously described [67], in accordance with European Academy of Allergy and Clinical Immunology (EAACI) guidelines [68].

Patients who met the clinical criteria for dupilumab prescription were selected by the Multidisciplinary Asthma Unit. The administration of dupilumab was initiated at 400 mg, followed by subsequent administrations of 200 mg every 2 weeks in the asthma group that was not receiving OCS at entry. In contrast, the asthma group that received OCS at entry and those with concomitant CRSwNP or N-ERD received an initial dose of 600 mg, followed by subsequent administrations of 300 mg every fortnight. The study was approved by the Clinical Research Ethics Committee (PI 2020 02 433), and all participants provided informed consent.

### 4.2. Study Variables and Clinical Measurements

Asthma Control Test (ACT) results, fractional exhaled nitric oxide (FeNO), and spirometric lung function parameters were obtained in all patients before and after 6 months of dupilumab treatment. The FEOS index [26,69] was calculated to assess asthma treatment response, while the Sino-Nasal Outcome Test (SNOT-22) was used to evaluate CRSwNP responses, with consideration of the SNOT-22 stratification proposed by Toma and Hopkins [29].

Furthermore, blood cell counts were conducted using an automatic counter (Beckman Coulter, Brea, CA, USA) and the MAXM A/L system (Beckman Coulter). Total serum IgE measurements were quantified using a fluoroenzyme immunoassay (ImmunoCap System, ThermoFisher Scientific, Waltham, MA, USA).

### 4.3. Transcriptomic RNA Sequencing

Two RNA-sequencing (RNAseq) transcriptomic analyses were conducted to investigate gene expression changes related to type 2 inflammatory disease (HCs (*n* = 30) vs. patients (*n* = 18)) and to dupilumab treatment (pre- vs. post-treatment; *n* = 18). The transcriptomic study was performed as previously described [41,42,70]. Briefly, total RNA was isolated from peripheral blood samples previously stored at −20 °C with RNA Later using the Ambion RiboPure™-Blood Kit (Thermo Fisher Scientific, Waltham, MA, USA). For RNA-seq analyses, 1 µg of RNA with an integrity value (RIN) > 8 was used. After removing globin and ribosomal RNA transcripts, the remaining RNA was cleaved to prepare RNA strand-specific libraries. The libraries were then clustered and sequenced using a high-throughput Illumina HiSeq 2500 (Illumina, San Diego, CA, USA).

### 4.4. Flow Cytometry Analysis

Peripheral blood samples were taken immediately before dupilumab treatment and six months later. Samples were collected in EDTA-coated tubes, and red blood cells were lysed before the flow cytometry analysis. Labeling of PTGDR2+ subpopulations of lymphocytes and basophils was done with a mixture of fluorophore-tagged monoclonal antibodies: CD3-FITC, CD123-APC, CD45-PerCP, and PTGDR2-PE. The samples were acquired using a Becton Dickinson (BD) FACSAria II cell sorter (BD Biosciences, Franklin Lakes, NJ, USA), and data were analyzed using FlowJo software (v10.9).

### 4.5. RNA-Seq Bioinformatics Analysis

In the bioinformatic analysis of transcriptomic data, FastQC [71] was used to assess the quality of raw reads. Sequencing reads were mapped to the human reference genome (GRCh38) using the TopHat2 software (version 2.1.1) [72]. Low-quality reads were removed using Picard Tools [73] and the unmapped or improperly paired reads were re-mapped using the BWA-MEM algorithm [74]. Gene and isoform prediction were estimated using the Cufflinks method [75]. The HTSeq software (v.0.6.0) [76] was used to calculate gene expression levels. Differential gene expression analysis between different conditions was performed on paired samples using DESeq2 (R package, version 1.46.0), following the standard workflow and normalization procedures implemented in the package documentation. DESeq2 is a widely used and well-established pipeline for RNA-seq differential expression analysis based on negative binomial generalized linear models. Statistical significance was assessed using the default DESeq2 framework, and *p*-values were adjusted for multiple testing according to the method implemented in the package documentation [77]. Genes with a log2 fold-change value ≤ −1.5 or ≥1.5 with a *p*-value (padj) corrected by Benjamini–Hochberg false discovery rate (FDR) <0.05 were considered as differently expressed genes (DEGs).

Given the heterogeneity of the patient samples, transcriptomic data were used to identify possible subgroups of patients with a similar genetic background within the initial population. A latent space analysis was performed using normalized values [27]. Data were further normalized using the derived rank matrix. Principal component analysis (PCA) was then applied, retaining components explaining at least 95% of the total variance. Subsequently, clustering analyses or specific homogeneity tests were conducted, as appropriate. To ensure this selection was unbiased, another unsupervised analysis was conducted across the entire cohort. All possible combinations were systematically evaluated, and differential expression analysis was performed using DESeq2, showing similar results [28]. The DEG list for each subset was evaluated using a scoring metric based on the number of genes meeting the following criteria: absolute log_2_ fold change > 1, *p*-value < 0.05, and multiple-testing-adjusted *p*-value (FDR). Correlations were performed between gene expression levels and peripheral blood immune cell counts obtained from complete blood count (CBC) with differential, including eosinophils, neutrophils, and total lymphocytes. To further explore the distinctiveness of the different patient subsets obtained, the complete set of hemogram and cytometry parameters was also evaluated in the study. Furthermore, potential interactions among selected proteins were examined using cluster analysis with the STRING software (version 12.0) [78]. Gene set enrichment analysis (GSEA) was performed to identify biological pathways and molecular signatures enriched among differentially expressed genes from the transcriptomic dataset using the WebGestalt online tool. For this, genes were ranked by log_2_ fold change (controls vs. asthmatic patients; pre-treatment vs. post-treatment), from lowest to highest.

### 4.6. Quantitative PCR Validation Analysis

The transcriptomic gene expression data of 23 genes were validated through independent quantitative PCR (qPCR) assays. Gene expression levels were determined in the 66 samples (18 samples pre- and post-treatment, along with 30 HCs). Supplementary Table S13 provides detailed information on the selected genes and individuals used to validate the results of each RNA-seq transcriptomic analysis. Relative expression by qPCR was carried out as previously described [67], using peripheral blood samples stored at −20 °C with RNA Later. In summary, total RNA was isolated using the Ambion RiboPure™-Blood Kit (Thermo Fisher Scientific, Waltham, MA, USA), followed by DNase treatment with Turbo DNAse (Ambion, Thermo Fisher Scientific, Waltham, MA, USA). Concentrations and RNA quality ratios were determined using a NanoDrop 1000 spectrophotometer (Thermo Fisher Scientific, Waltham, MA, USA). Reverse transcription was carried out using the Superscript III First-Strand Synthesis System for RT-PCR (Invitrogen, Thermo Fisher Scientific, Waltham, MA, USA). Relative qPCR was carried out using the LightCycler480^®^ Instrument and SYBR Green I Master (Roche, Basel, Switzerland). The cycling conditions consisted of an initial pre-incubation step at 95 °C for 5 min, followed by 45 amplification cycles including denaturation at 95 °C for 10 s, annealing at 60 °C for 10 s, and extension at 72 °C for 10 s. A melting curve analysis was subsequently performed to verify amplification specificity (95 °C for 5 s, 65 °C for 1 min, followed by continuous heating to 97 °C, and a final cooling step was carried out at 40 °C for 30 s). The comparative cycle threshold (CT) method (2^−ΔΔCt^) [79] was employed for expression analysis. Each sample was measured in triplicate, with *GAPDH* as the reference gene and following MIQE guidelines [80]. Primer sequences were either commercially obtained or designed de novo using the NCBI Primer-BLAST tool (access date 2025) [81]. Primer specificity was verified in silico using BLAST analysis to ensure unique target amplification and to avoid off-target binding. All primer pairs were designed to meet standard qPCR criteria, including amplicon length of 20–25 bp, annealing temperatures close to 60 °C, GC content of 40–60%, and avoidance of secondary structures such as hairpins, self-dimers, and cross-dimers. Primer performance was experimentally validated using standard curves generated from serial dilutions of cDNA (100 ng to 0.01 ng). Amplification efficiencies were calculated from the slope of the standard curves, and all primer sets showed efficiencies >80%. The primer sequences used for the amplification of each gene are shown in Supplementary Table S14.

### 4.7. Statistical Analysis

Statistical analyses were performed using IBM SPSS Statistics Software version 30 (IBM, Armonk, NY, USA), and graphs were plotted using GraphPad Prism version 6 (San Diego, CA, USA). The Kolmogorov–Smirnov Z-test was employed to determine the normality of the distribution.

Between groups (HCs vs. patients and SR vs. non-SR) continuous variables were compared using the Mann–Whitney U test, while categorical variables were analyzed with the chi-square test. Data analysis was performed using pairwise comparison by the Wilcoxon test. Associations between patient characteristics (age, sex, atopy, asthma exacerbations in the previous year, use of oral corticosteroids or montelukast before therapy, baseline SNOT-22 score, and smoking history) and treatment response were analyzed. Correlations among continuous variables were assessed using Spearman’s correlation coefficients. Baseline gene expression levels were compared between super-responders and non-super-responders using the Mann–Whitney U test for independent samples. No multivariate predictive model or machine learning approach was applied. For exploratory purposes, receiver operating characteristic (ROC) analysis was used to assess the diagnostic performance of the potential biomarkers. To evaluate each biomarker’s discriminatory capacity, univariate ROC curves were constructed. The ROC curves were constructed based on the ranking of biomarker values and the evaluation of all possible cut-off points to classify subjects into their actual groups correctly. The area under the curve (AUC) was estimated using the non-parametric Mann–Whitney U test, which corresponds to the probability that an individual in the positive group has a higher value than one in the negative group. The threshold for statistical significance was set at *p* < 0.05 for all analyses.

## 5. Conclusions

In summary, this study characterizes systemic transcriptomic changes associated with severe asthma and IL-4/IL-13 blockade. We identify two molecular endotypes underlying differential transcriptional responses to dupilumab and propose candidate biomarkers for treatment stratification. In the CRSwNP patients’ response, DIXDC1 emerges as a biomarker of non-SR. Our findings indicate that dupilumab drives a coordinated shift from inflammatory activation toward immune regulation, tissue repair, and metabolic normalization, reflecting immune recalibration. Importantly, these transcriptomic changes parallel the clinical improvement observed in treated patients. These results underscore the power of peripheral transcriptomic profiling to enable precise patient stratification and guide personalized therapy in severe asthma.

## Figures and Tables

**Table 1:** Demographic and clinical characteristics of healthy non-asthmatic controls and patients.

	Control Group (*n* = 30)	Patients (*n* = 18)		
		Baseline (T0)	After Six Months of Treatment (T6)	*p*-Value
Male sex (%)	11 (36.7%)	11 (61.1%) 0.100 †	–	–
Age	57.9 ± 19.2	52.2 ± 10.7 0.133 †	–	–
BMI	N/A	26.4 ± 3.5	–	–
Ever-smoker (%)	N/A	10 (55.6%)	–	–
Atopy (%)	0 (0%)	9 (50.0%)	–	–
Severe asthma (%)	0 (0%)	18 (100%)	–	–
Polyposis (%)	0 (0%)	6 (33.3%)	–	–
N-ERD (%)	0 (0%)	12 (66.7%)	–	–
OCS use (%)	N/A	5 (27.8%)	3 (16.7%)	0.002 *
Exacerbation/year	N/A	2.2 ± 3.9	0.0 ± 0.0	0.007 *
ACT	N/A	17.3 ± 6.4	23.0 ± 3.1	0.009 *
FEV1 (mL)	N/A	2516 ± 878	3080 ± 985	0.008 *
FEV1 (%)	N/A	76.8 ± 18.7	96.3 ± 19.4	0.006 *
FeNO (ppb)	N/A	60.9 ± 42.8	33.5 ± 28.2	0.023 *
SNOT-22	N/A	56.4 ± 23.8	20.2 ± 17.2	0.001 *
Serum IgE (kU/L)	74 ± 107	537 ± 1158 <0.001 †	216 ± 528	<0.001 *
Eosinophils/µL	133 ± 84	514 ± 514 0.019 †	1049 ± 656	0.011 *

Data are presented as the mean ± Standard Deviation or frequency (percentage, %) with the corresponding *p*-value. ACT, Asthma Control Test; BMI, Body mass index; FeNO, Fractional exhaled nitric oxide; FEV1, Exacerbations, Oral corticosteroids, Symptoms score [26]; FEV1, Forced expiratory volume in 1 s; *n*, number of samples; N-ERD, Non-steroidal anti-inflammatory drugs intolerance exacerbated respiratory disease; OCS use (%) indicates the proportion of patients receiving oral corticosteroids at baseline (T0); SNOT-22, Sino-Nasal Outcome Test-22; *p*-value †, Mann–Whitney *p*-value/Chi-square *p*-value (comparison between controls and dupilumab patients at baseline); *p*-value *, Wilcoxon test *p*-value/Chi-square *p*-value (comparison between baseline and six months after dupilumab treatment).

**Table 2:** Classification of the response to treatment with dupilumab.

ID	Diagnostic	Previous Biological Treatment	Asthma		Polyposis		
			FEOS Index	Response	SNOT-22 PRE	SNOT-22 POST	Response
1 ♈	N-ERD	Yes	100%	SR	81	0	SR
2 ♈	N-ERD	Yes	100%	SR	77	32	Non-SR
3 ♈	Asthma and CRSwNP	No	100%	SR	Non-available	Non-available	Non-available
4 ♈	N-ERD	Yes	85%	Non-SR	Non-available	30	Non-SR
5 ♈	N-ERD	Yes	100%	SR	62	8	Non-SR
6 ♈	Asthma and CRSwNP	Yes	52%	Non-SR	23	Non-available	Non-available
7 ♈	Asthma and CRSwNP	No	100%	SR	28	1	SR
8 ♈	Asthma and CRSwNP	No	100%	SR	59	19	Non-SR
9 ♈	N-ERD	Yes	67%	Non-SR	81	55	Non-SR
10 ♈	N-ERD	Yes	100%	SR	16	8	Non-SR
11 #	N-ERD	No	100%	SR	55	14	Non-SR
12 #	N-ERD	No	100%	SR	71	12	Non-SR
13 #	N-ERD	No	100%	SR	83	37	Non-SR
14 #	N-ERD	Yes	78%	Non-SR	40	15	Non-SR
15 #	N-ERD	No	100%	SR	93	32	Non-SR
16 #	Asthma and CRSwNP	Yes	85%	Non-SR	52	50	Non-SR
17 #	Asthma and CRSwNP	No	100%	SR	31	5	SR
18 #	N-ERD	Yes	100%	SR	50	5	SR

CRSwNP, Chronic rhinosinusitis with nasal polyposis; N-ERD, Non-steroidal anti-inflammatory drugs exacerbated respiratory disease; Non-SR, Non-super-responder; SNOT-22, Sino-Nasal Outcome Test-22; SR, Super-responder; ♈, G1 samples; #, G2 samples.

**Table 3:** Demographic and clinical characteristics of subgroups of patients from the transcriptomic study, including cytometry and hemogram differences considered for subgroup classification.

	Transcriptomic Group G0 (*n* = 18)			Subgroups						
				Subgroup G1 (*n* = 10)			Subgroup G2 (*n* = 8)			
	T0	T6	*p*-Value	T0	T6	*p*-Value	T0	T6	*p*-Value	Ratio *p*-Value *
**Male sex (%)**	11 (61.1%)			6 (60.0%)			5 (62.5%)			ns
**Age**	52.2 ± 10.7			51.4 ± 9.1			53.3 ± 13.0			ns
**BMI**	26.4 ± 3.5			25.5 ± 2.7			27.9 ± 4.2			ns
**Ever-smoker (%)**	10 (55.6%)			6 (60.0%)			4 (50.0%)			ns
**Atopy (%)**	9 (50.0%)			6 (60.0%)			3 (37.5%)			ns
**Asthma and Polyposis (%)**	6 (33.3%)			4 (40.0%)			2 (25.0%)			ns
**N-ERD (%)**	12 (66.7%)			6 (60.0%)			6 (75.0%)			ns
**OCS use (%)**	5 (27.8%)	3 (16.7%)	0.500	5 (50.0%)	3 (30.0%)	0.500	0 (0%)	0 (0%)	1.000	0.043
**Exacerbation/year**	2.2 ± 3.9	0.0 ± 0.0	0.007	3.5 ± 4.9	0.0 ± 0.0	0.027	0.5 ± 0.8	0.0 ± 0.0	0.102	ns
**ACT**	17.3 ± 6.4	23.0 ± 3.1	0.009	15.3 ± 6.3	22.2 ± 3.9	0.034	19.9 ± 6.1	24.0 ± 1.2	0.138	ns
**FEV1 (mL)**	2516 ± 878	3080 ± 985	0.008	2444 ± 682	3202 ± 835	0.047	2606 ± 1121	2928 ± 1188	0.093	ns
**FEV1 (%)**	76.8 ± 18.7	96.3 ± 19.4	0.006	76.4 ± 19.5	100.0 ± 20.7	0.047	77.3 ± 19.0	91.6 ± 17.7	0.036	ns
**FENO (ppb)**	60.9 ± 42.8	33.5 ± 28.2	0.023	77.3 ± 47.4	31.8 ± 14.9	0.021	39.7 ± 25.4	35.9 ± 42.1	0.753	ns
**SNOT-22**	56.4 ± 23.8	20.2 ± 17.2	<0.001	53.4 ± 27.1	19.1 ± 18.9	0.018	59.4 ± 21.3	21.3 ± 16.5	0.012	ns
**Serum IgE (kU/L)**	537 ± 1158	216 ± 528	<0.001	852 ± 1507	350 ± 692	0.005	144 ± 137	48 ± 46	0.012	ns
**Eosinophils/µL**	514 ± 514	1049 ± 656	0.011	327 ± 450	1352 ± 665	0.005	749 ± 517	671 ± 426	0.866	0.037 *
**Eosinophil Ratio_T6/T0**	2.04 ± 1.28			4.13 ± 1.48			0.90 ± 0.82			<0.001 *
**Cells (PTGDR2+_T0) #**	5319 ± 3636	11,650 ± 7085	0.008	3320 ± 3220	14,285 ± 4102	0.016	7068 ± 3173	9344 ± 8540	0.945	0.47 0.056 *
**Basophils (CD45+_T6)#**	620 ± 516	1110 ± 638	0.019	615 ± 628	1480 ± 665	0.016	624 ± 441	786 ± 422	0.313	1.88 0.039 *
**Lymphocytes (CD3+CD45+PTGDR2+_T6) #**	95 ± 74	118 ± 65	0.150	128 ± 69	169 ± 42	0.297	67 ± 69	74 ± 47	0.674	2.30 0.001 *

Data are shown as the mean ± Standard Deviation or frequency (percentage, %). ACT, Asthma Control Test; BMI, Body mass index; FENO, Fractional exhaled nitric oxide; FEV1, Forced expiratory volume in 1 s; *n*, number of samples; G0, total cohort; G1, subgroup 1; G2, subgroup 2; N-ERD, Non-steroidal anti-inflammatory drugs intolerance exacerbated respiratory disease; OCS, Oral corticosteroids; SNOT-22, Sino-Nasal Outcome Test-22; T0, Before treatment; T6, 6 months after treatment; #, Cytometry samples; *p*-value, Wilcoxon Test *p*-value for paired dichotomous variables was obtained using McNemar’s test; * *p*-value, Normality was tested with Shapiro–Wilk test and the *t*-test or Mann–Whitney test was used for calculate *p*-value between G1 vs. G2; ns, Non-significative.

**Table 4:** Effect sizes and confidence intervals for differences in gene expressions between controls and patients (T0 expression) and between T0 and T6.

Gene	Control, Median (IQR)	P-G0-T0, Median (IQR) (*p*-Value C vs. T0-G0)	P-G1-T0, Median (IQR) (*p*-Value C vs. T0-G1)	P-G2-T0, Median (IQR) (*p*-Value C vs. T0-G2)	P-G0-T6, Median (IQR) (*p*-Value T0 vs. T6-G0)	P-G1-T6, Median (IQR) (*p*-Value T0 vs. T6-G1)	P-G2-T6, Median (IQR) (*p*-Value T0 vs. T6-G2)
*ABCC1*	1.332	3.678	3.056	4.546	4.927	4.927	4.939
	(0.456, 1.983)	(2.491, 5.036)	(2.099, 4.539)	(3.676, 5.095)	(3.949, 5.939)	(3.949, 5.604)	(3.914, 6.988)
		<0.001 *	<0.001 *	<0.001 *	0.094 †	0.169 †	0.575 †
*ABTB2*	0.871	2.85	1.514	4.781	6.357	6.927	4.676
	(0.659, 1.654)	(1.109, 5.046)	(0.881, 2.623)	(4.072, 5.488)	(4.047, 7.912)	(5.007, 7.946)	(2.625, 8.169)
		<0.001 *	0.126 *	<0.001 *	0.014 †	0.009 †	1.000 †
*CLC*	1.190	3.701	1.328	8.407	7.148	8.371	6.116
	(0.489, 2.129)	(0.737, 9.619)	(0.433, 4.285)	(4.258, 11.011)	(3.099, 14.411)	(3.590, 14.427)	(1.950, 18.999)
		0.003 *	0.532 *	<0.001 *	0.043 †	0.047 †	0.779 †
*CYP4F12*	1.049	6.733	2.386	11.438	13.581	17.137	10.817
	(0.633, 1.610)	(1.997, 14.799)	(0.776, 14.799)	(6.706, 25.311)	(8.330, 25.490)	(11.436, 28.387)	(6.990, 13.714)
		<0.001 *	0.049 *	<0.001 *	0.064 †	0.013 †	1.000 †
*CYSLTR2*	1.091	1.961	0.895	2.989	4.331	4.615	3.951
	(0.822, 1.403)	(0.633, 3.897)	(0.450, 2.568)	(2.097, 4.240)	(2.031, 6.347)	(2.876, 7.249)	(1.739, 5.933)
		0.016 *	0.827 *	<0.001 *	0.018 †	0.028 †	0.575 †
*DIXDC1*	1.000	1.219	1.219	1.176	1.243	1.260	1.243
	(0.580, 1.653)	(0.802, 1.473)	(0.694, 1.526)	(0.864, 1.453)	(0.850, 2.085)	(0.850, 2.431)	(0.775, 1.717)
		0.375 *	0.595 *	0.371 *	0.647 †	0.445 †	0.575 †
*FBN1*	1.099	6.186	3.442	7.557	9.562	10.213	7.972
	(0.589, 1.520)	(3.298, 8.185)	(0.573, 6.697)	(6.147, 9.668)	(6.261, 12.748)	(7.527, 13.364)	(3.989, 13.091)
		<0.001 *	0.023 *	<0.001 *	0.022 †	0.007 †	1.000 †
*H3C13*	1.209	1.515	1.179	2.053	1.239	1.367	1.058
	(0.618, 1.760)	(1.051, 2.554)	(0.770, 2.554)	(1.234, 3.860)	(1.011, 1.944)	(1.105, 1.944)	(0.842, 2.265)
		0.048 *	0.453 *	0.013 *	0.557 †	0.878 †	0.401 †
*H3P6*	1.081	1.381	1.134	2.017	1.450	1.241	1.512
	(0.480, 2.270)	(0.857, 2.116)	(0.736, 1.440)	(1.097, 2.412)	(0.859, 1.844)	(0.859, 1.844)	(0.758, 2.085)
		0.639 *	0.755 *	0.252 *	0.679 †	0.285 †	0.484 †
*H4C11*	0.894	1.281	1.263	1.478	1.300	1.605	1.136
	(0.761, 1.437)	(0.946, 1.953)	(1.028, 1.734)	(0.783, 2.113)	(0.893, 1.949)	(0.981, 2.046)	(0.568, 1.861)
		0.106 *	0.151 *	0.283 *	0.811 †	0.386 †	0.674 †
*H4C4*	0.822	0.365	0.365	0.709	0.469	0.497	0.386
	(0.552, 1.770)	(0.176, 1.582)	(0.218, 0.988)	(0.090, 2.240)	(0.216, 0.908)	(0.259, 0.816)	(0.125, 0.957)
		0.023 *	0.010 *	0.371 *	0.420 †	0.721 †	0.484 †
*HRH4*	1.050	3.154	1.106	4.810	6.113	7.758	4.678
	(0.685, 1.427)	(0.969, 5.100)	(0.495, 3.095)	(3.628, 8.641)	(3.324, 11.777)	(4.377, 13.246)	(1.782, 7.483)
		0.014 *	0.876 *	<0.001 *	0.122 †	0.017 †	0.401 †
*IKZF2*	1.061	1.957	1.623	2.374	3.862	3.337	4.161
	(0.563, 1.396)	(1.473, 3.728)	(1.283, 2.739)	(1.762, 4.820)	(2.383, 5.106)	(2.679, 5.186)	(1.940, 5.103)
		0.002 *	0.029 *	0.009 *	0.018 †	0.037 †	0.263 †
*IL5Ra*	1.137	6.432	0.725	11.055	13.917	19.318	9.782
	(0.568, 1.683)	(0.634, 13.494)	(0.538, 6.732)	(6.977, 16.385)	(6.447, 22.249)	(6.447, 23.955)	(6.169, 21.368)
		0.007 *	0.839 *	<0.001 *	0.012 †	0.007 †	0.674 †
*LGALS9*	1.126	1.396	1.224	1.805	1.756	1.921	1.491
	(0.645, 1.516)	(0.817, 2.162)	(0.724, 1.957)	(0.848, 4.309)	(1.212, 2.613)	(1.212, 3.051)	(1.217, 2.550)
		0.113 *	0.357 *	0.107 *	0.267 †	0.059 †	0.779 †
*PTGDR2*	0.929	3.310	1.045	5.656	6.516	6.607	6.324
	(0.612, 1.730)	(0.744, 5.926)	(0.289, 4.036)	(3.092, 7.034)	(4.139, 8.575)	(4.139, 8.414)	(2.795, 11.575)
		0.017 *	0.851 *	<0.001 *	0.018 †	0.028 †	0.484 †
*RAB44*	1.007	3.978	2.509	4.784	8.376	9.908	4.613
	(0.643, 1.343)	(2.026, 5.332)	(0.913, 5.118)	(3.576, 11.936)	(4.333, 10.719)	(7.095, 12.052)	(1.647, 8.058)
		<0.001 *	0.011 *	<0.001 *	0.112 †	0.017 †	0.401 †
*SETD1B*	1.138	2.360	1.225	4.543	2.796	3.927	2.751
	(0.643, 1.811)	(1.141, 4.160)	(0.920, 2.303)	(2.900, 9.184)	(2.254, 5.777)	(2.323, 7.021)	(2.107, 3.603)
		0.003 *	0.357 *	<0.001 *	0.557 †	0.009 †	0.036 †
*SIRPB1*	0.913	0.562	0.606	0.546	0.496	0.465	0.727
	(0.784, 1.370)	(0.248, 1.250)	(0.194, 1.557)	(0.423, 1.053)	(0.435, 0.871)	(0.435, 0.759)	(0.277, 0.951)
		0.015 *	0.151 *	0.015 *	0.811 †	0.878 †	0.674 †
*SMPD3*	1.131	6.959	0.914	11.617	16.158	18.626	13.469
	(0.628, 1.563)	(0.641, 16.168)	(0.549, 10.67)	(6.654, 16.393)	(4.786, 23.583)	(8.868, 24.718)	(2.834, 22.447)
		0.005 *	0.685 *	<0.001 *	0.022 †	0.028 †	0.484 †
*SRGAP3*	1.197	3.244	1.083	4.850	8.896	9.014	7.087
	(0.538, 1.492)	(1.012, 5.500)	(0.931, 5.279)	(2.846, 8.843)	(3.502, 11.118)	(5.364, 13.040)	(2.659, 10.304)
		0.010 *	0.382 *	0.001 *	0.020 †	0.022 †	0.401 †
*TRPC6*	1.214	1.366	1.018	2.505	2.671	2.671	2.734
	(0.605, 1.570)	(0.882, 3.255)	(0.423, 1.480)	(1.332, 7.004)	(1.992, 5.524)	(1.925, 4.625)	(1.809, 6.240)
		0.229 *	0.390 *	0.003 *	0.094 †	0.047 †	0.889 †
*VSTM1*	1.067	1.003	0.653	2.050	2.216	2.322	2.216
	(0.743, 1.386)	(0.540, 2.221)	(0.343, 1.100)	(1.106, 4.720)	(1.329, 5.535)	(1.578, 5.952)	(1.295, 5.091)
		0.694 *	0.075 *	0.007 *	0.145 †	0.037 †	0.779 †

C, Non-asthmatic control; G0, Transcriptomic group (*n* = 18); G1, Subgroup 1 (*n* = 10); G2, Subgroup 2 (*n* = 8); IQR, Interquartile range; * Mann–Whitney *p*-value (comparison between healthy control vs. asthmatic patients): †, Wilcoxon *p*-value (comparison between pre- vs. post- dupilumab treatment).

## Data Availability

The transcriptomic data supporting this study’s findings are available in the Sequence Read Archive (NCBI) at https://www.ncbi.nlm.nih.gov/sra (accessed on 15 December 2025), reference number PRJNA1416005.

## Supplementary Materials

The following supporting information can be downloaded at: https://www.mdpi.com/article/10.3390/ijms27125266/s1.

## Abbreviations

The following abbreviations are used in this manuscript:

ACT	Asthma Control Test
AD	Atopic Dermatitis
AUC	Area Under the Curve
ATS	American Thoracic Society
COPD	Chronic Obstructive Pulmonary Disease
CRSwNP	Chronic Rhinosinusitis with Nasal Polyposis
DEGs	Differentially Expressed Genes
EAACI	European Academy of Allergy and Clinical Immunology
EDTA	Ethylenediaminetetraacetic Acid
EoE	Eosinophilic Esophagitis
FDA	Food and Drug Administration
FDR	False Discovery Rate
EPOS	European Position Paper on Rhinosinusitis and Nasal Polyps
ERS	European Respiratory Society
FeNO	Fractional Exhaled Nitric Oxide
FEOS	Forced Expiratory Obstruction Score
FEV_1_	Forced Expiratory Volume in 1 Second
GEMA	Spanish Guide for the Management of Asthma
GINA	Global Initiative for Asthma
GO	Gene Ontology
GRCh38	Genome Reference Consortium Human Build 38
GSEA	Gene Set Enrichment Analysis
HCs	Healthy Controls
IgE	Immunoglobulin E
IL	Interleukin
IL-4Rα	Interleukin-4 Receptor Alpha
N-ERD	Non-Steroidal Anti-Inflammatory Drug Exacerbated Respiratory Disease
NCBI	National Center for Biotechnology Information
NSAID	Non-Steroidal Anti-Inflammatory Drug
OCS	Oral Corticosteroid
PBMC	Peripheral Blood Mononuclear Cell
PCA	Principal Component Analysis
QoL	Quality of Life
PCR	Polymerase Chain Reaction
qPCR	Quantitative Polymerase Chain Reaction
RNA	Ribonucleic Acid
RNA-seq	Ribonucleic Acid Sequencing
ROC	Receiver Operating Characteristic
SNOT-22	Sino-Nasal Outcome Test-22
SR	Super-Response
STRING	Search Tool for the Retrieval of Interacting Genes
Th2	T Helper 2
Treg	T Regulatory
