# Supplementary material for: Unsupervised Machine-Learning-Based Endotype Discovery Using Iterative Resampling in Dupilumab-Treated Patients
Source: Int J Mol Sci. 2026 Jun 10;27(12):5266. doi: 10.3390/ijms27125266 (PMC13300600; doi:10.3390/ijms27125266)
Supplement: Supplementary file 1 [file ijms-27-05266-s001.zip › 3_Supplementary Figures Dupilumab - IJMS.pdf]

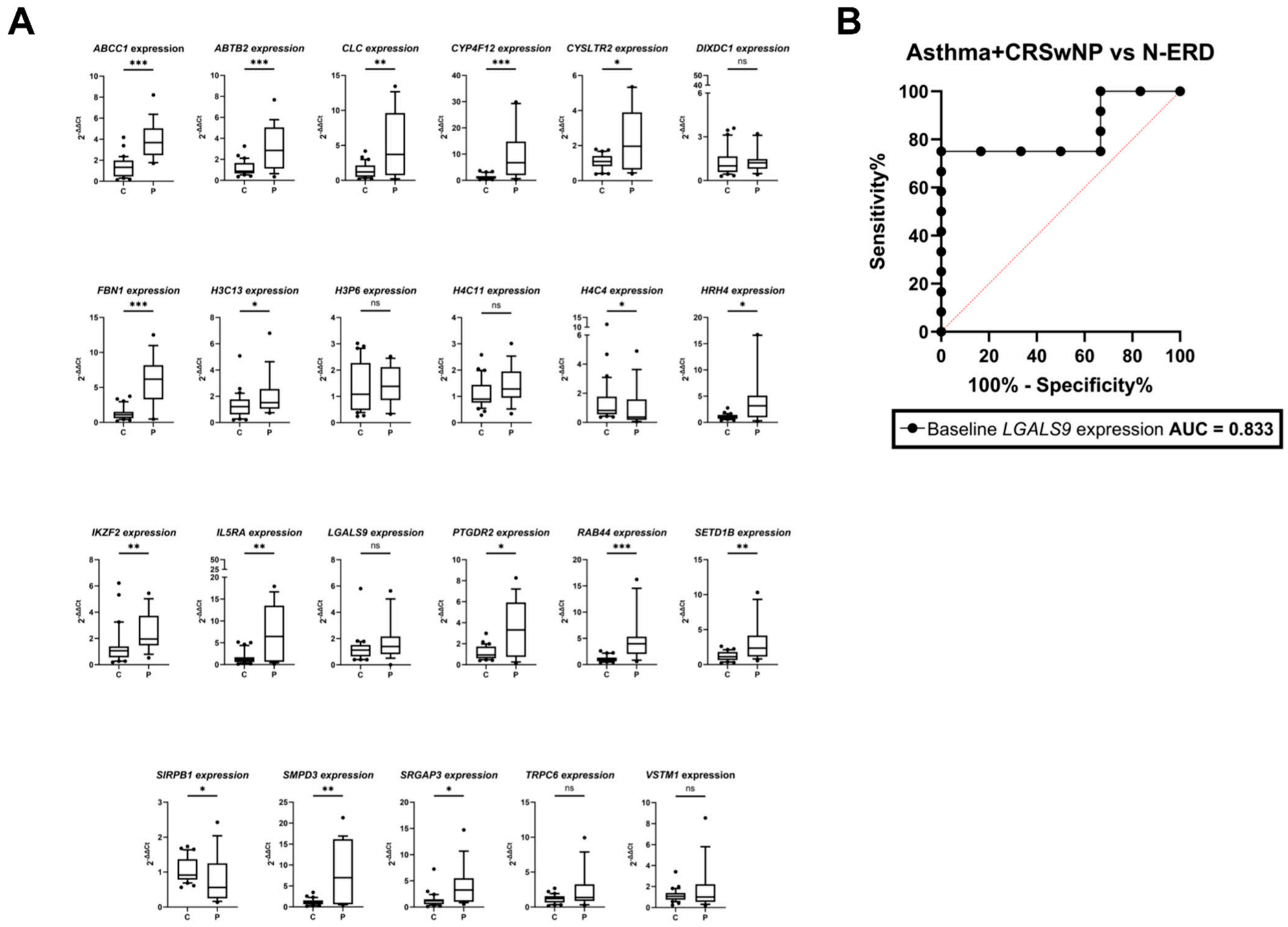

Figure S1. Gene expression changes between non-asthmatic controls and asthmatic patients. A) *ABCC1, ABTB2, CLC, CYP4F12, CYSLTR2, DIXDC1, FBN1, H3C13, H3P6, H4C11, H4C4, HRH4, IKZF2, IL5Ra, LGALS9, PTGDR2, RAB44, SETD1B, SIRPB1, SMPD3, SRGAP3, TRPC6* and *VSTM1* mRNA levels measured by qPCR between non-asthmatic controls and asthmatic patients (ns = non-significative; *, $p < 0.05$; **, $p < 0.010$; ***, $p < 0.001$; Mann-Whitney test). B) ROC curve illustrating the predictive value of baseline *LGALS9* expression for classifying patients as asthmatics with CRSwNP or N-ERD.

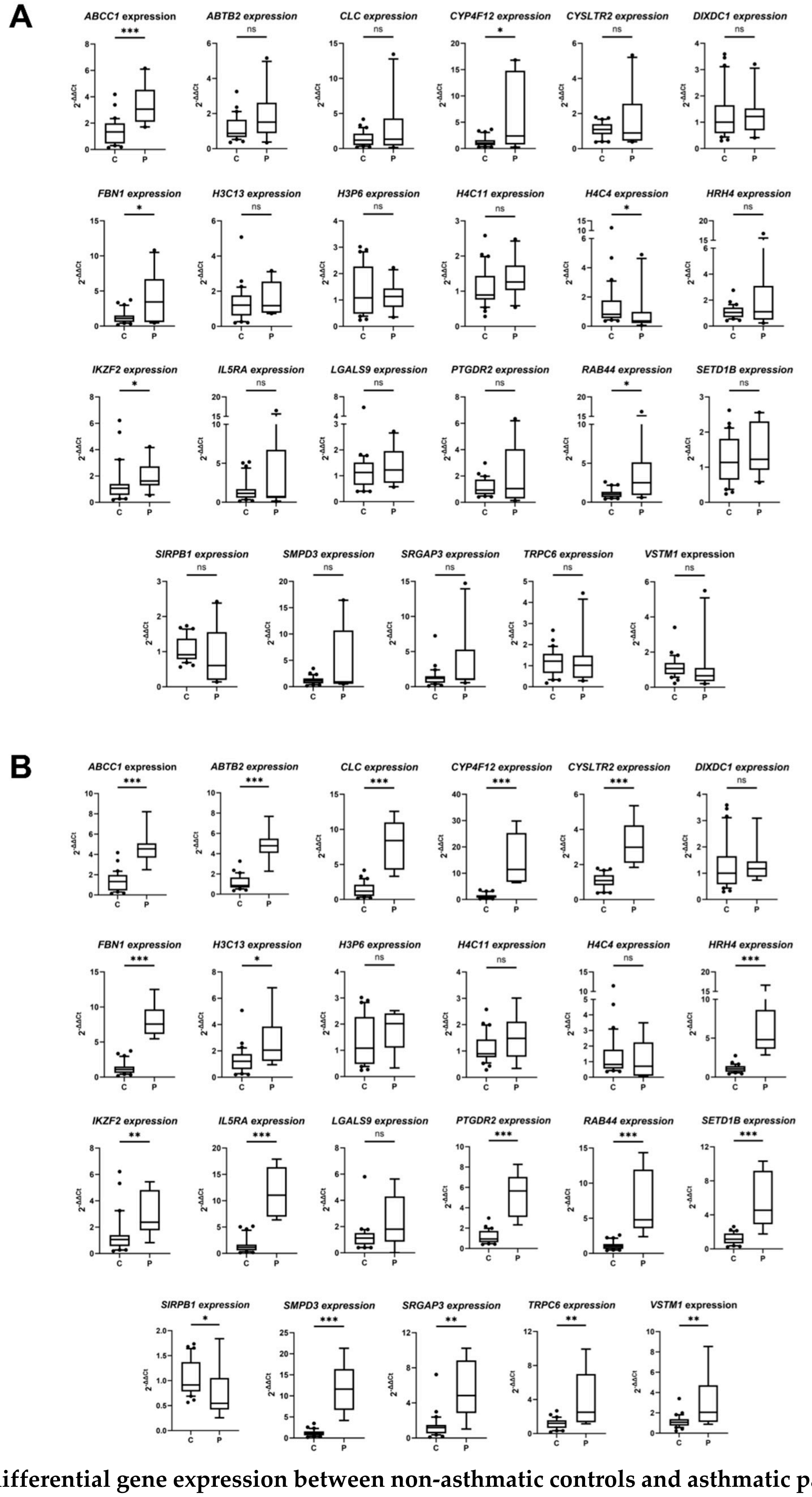

Figure S2. Baseline differential gene expression between non-asthmatic controls and asthmatic patients. A) Baseline gene expression levels were measured by qPCR comparing non-asthmatic controls with asthmatics in the G1 Subgroup of patients (n=10). B) Baseline gene expression levels were measured by qPCR comparing non-asthmatic controls with asthmatics in the G2 Subgroup of patients (n=8). (ns = non-significative; *, $p < 0.05$; **, $p < 0.01$, ***, $p < 0.001$, Mann-Whitney test).

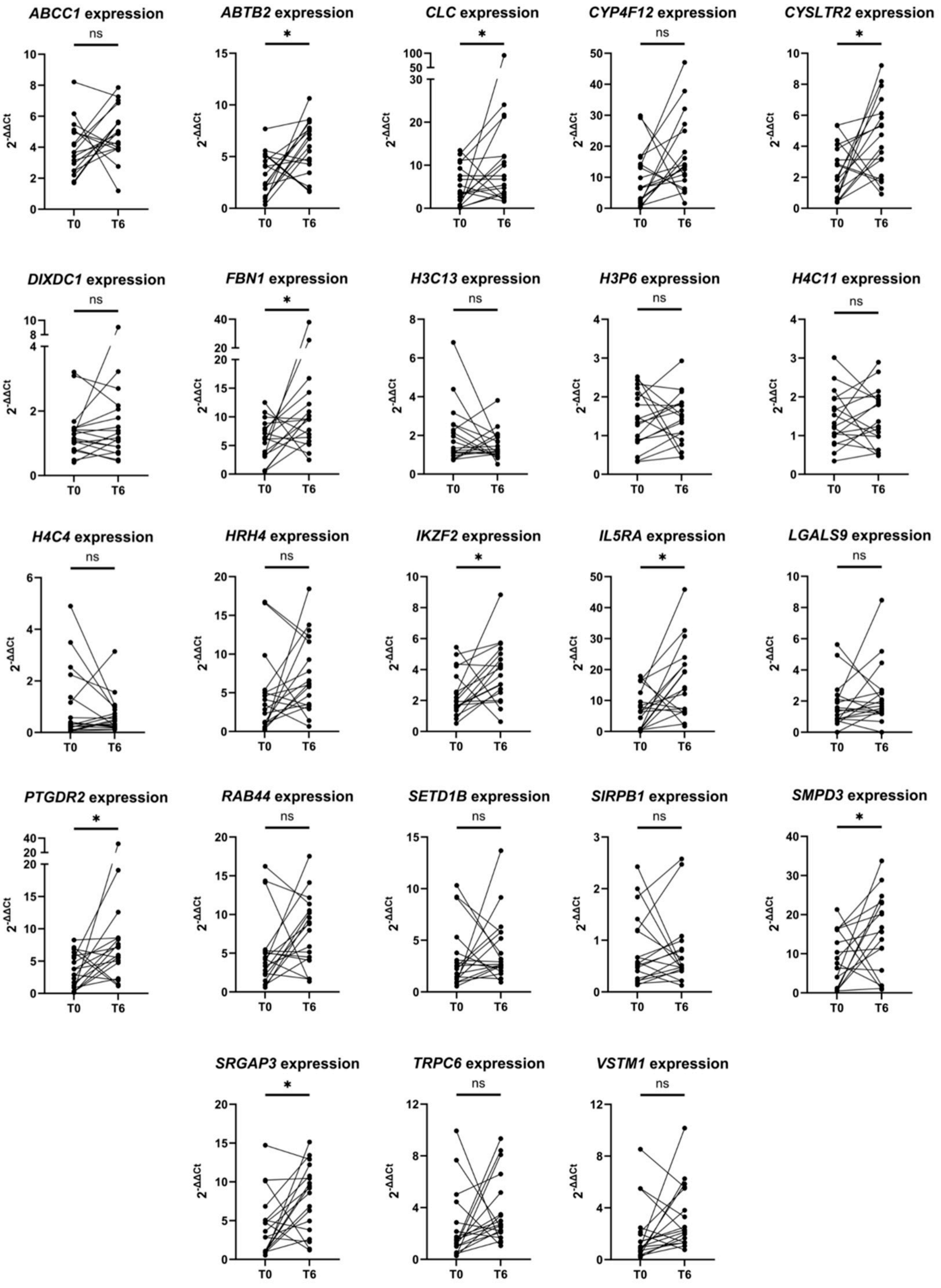

Figure S3. Gene expression changes between asthmatic patients before and 6 months after dupilumab treatment. *ABCC1, ABTB2, CLC, CYP4F12, CYSLTR2, DIXDC1, FBN1, H3C13, H3P6, H4C11, H4C4, HRH4, IKZF2, IL5Ra, LGALS9, PTGDR2, RAB44, SETD1B, SIRPB1, SMPD3, SRGAP3, TRPC6* and *VSTM1* mRNA levels measured by qPCR at baseline and after six months of dupilumab treatment in the study cohort (G0) (ns = non-significative; *****, $p < 0.05$; Wilcoxon test analysis).

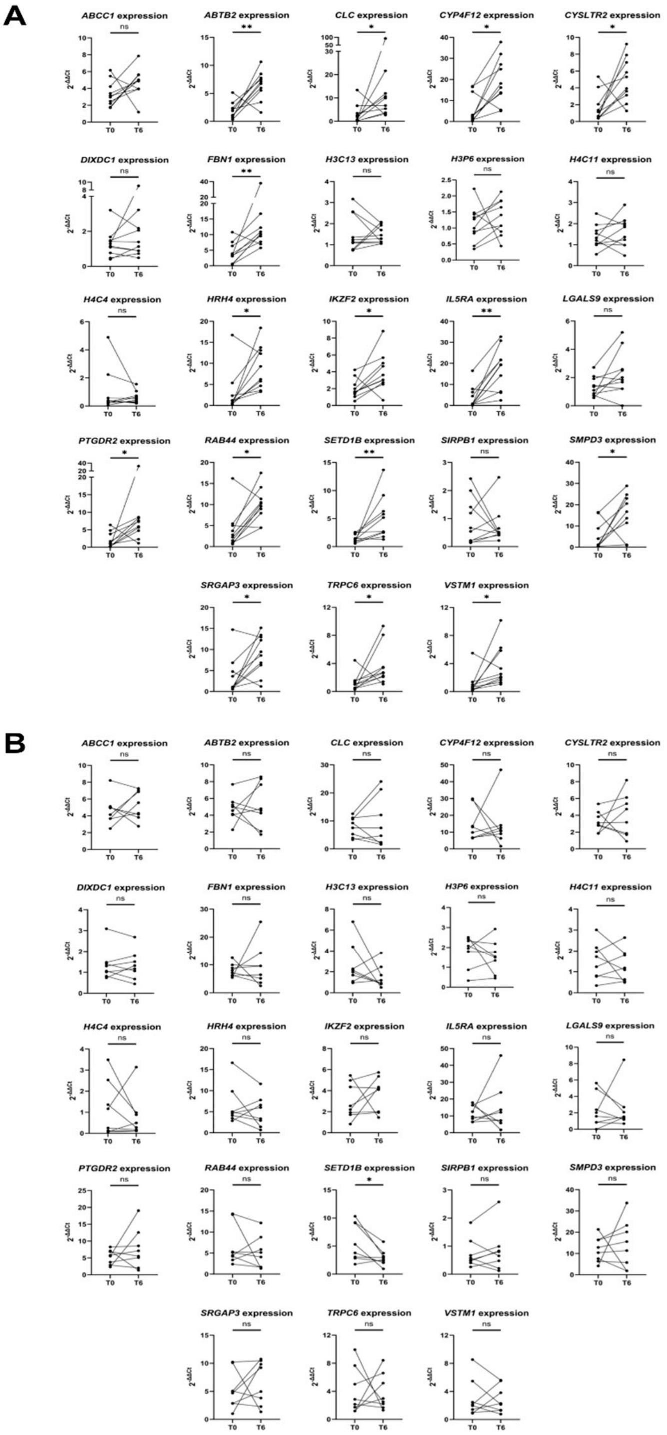

Figure S4. Gene expression changes between asthmatic patients before and 6 months after dupilumab treatment. A) *ABCC1, ABTB2, CLC, CYP4F12, CYSLTR2, DIXDC1, FBN1, H3C13, H3P6, H4C11, H4C4, HRH4, IKZF2, IL5Ra, LGALS9, PTGDR2, RAB44, SETD1B, SIRPB1, SMPD3, SRGAP3, TRPC6* and *VSTM1* mRNA levels measured by qPCR at baseline and after six months of dupilumab treatment in G1 Subgroup. B) *ABCC1, ABTB2, CLC, CYP4F12, CYSLTR2, DIXDC1, FBN1, H3C13, H3P6, H4C11, H4C4, HRH4, IKZF2, IL5Ra, LGALS9, PTGDR2, RAB44, SETD1B, SIRPB1, SMPD3, SRGAP3, TRPC6* and *VSTM1* mRNA levels measured by qPCR at baseline and after six months of dupilumab treatment in the G2 Subgroup. (ns = non-significative; *, p < 0.05; **, p < 0.010; Wilcoxon test analysis).
